# Supplementary figures and images for: Comparative Analysis of Bacterial Cellulose Membranes Synthesized by Chosen Komagataeibacter Strains and Their Application Potential
Source: Int J Mol Sci. 2022 Mar 21;23(6):3391. doi: 10.3390/ijms23063391 (PMC8950309; doi:10.3390/ijms23063391)

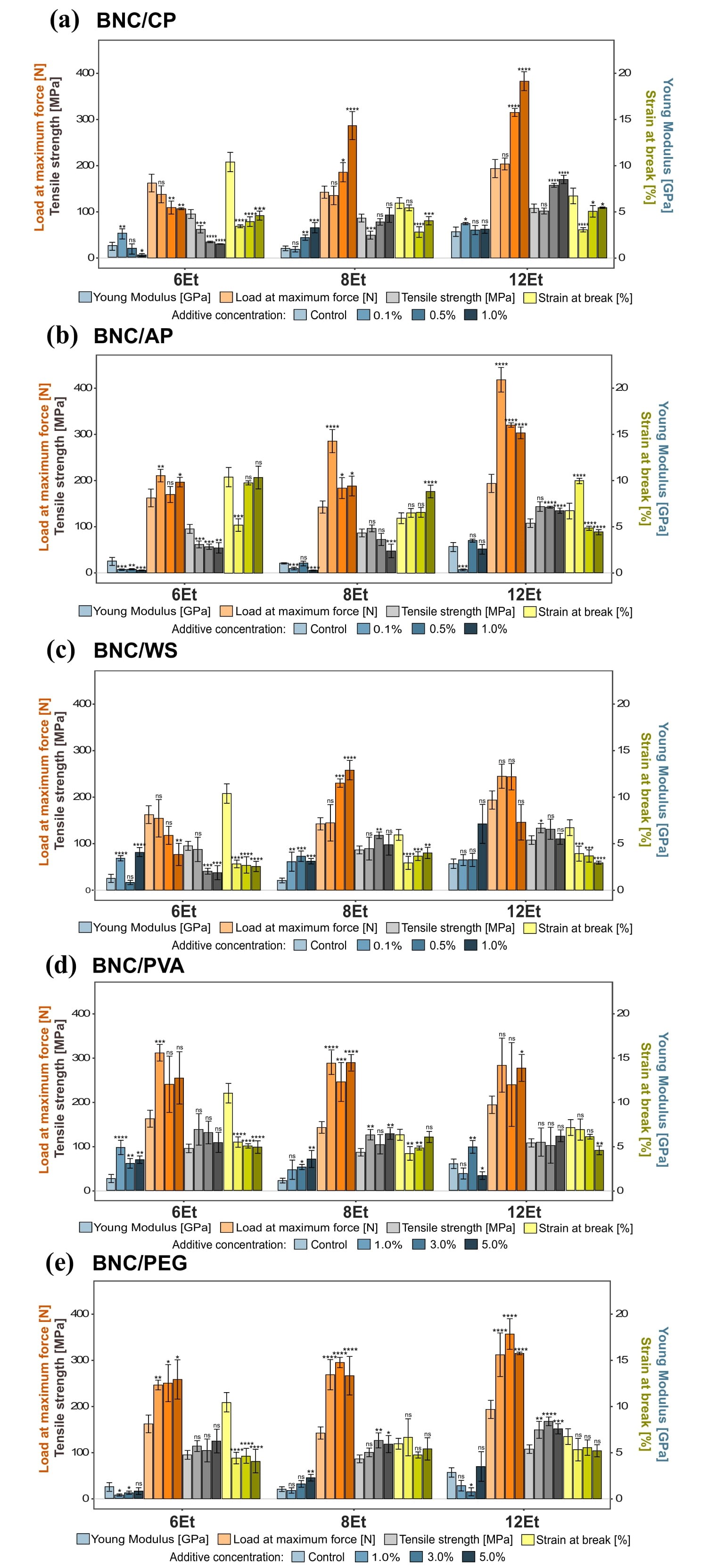

Supplement: Supplementary file 1 [file ijms-23-03391-s001.zip › Figure S1.jpeg]

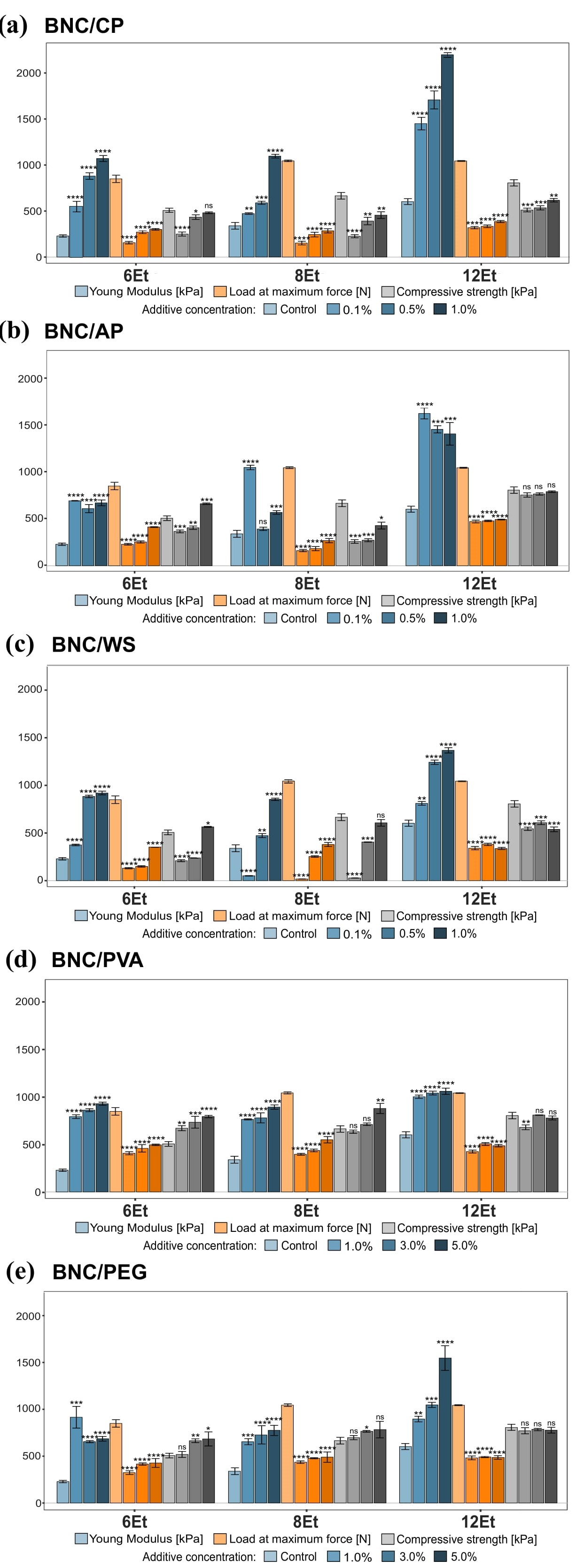

Supplement: Supplementary file 1 [file ijms-23-03391-s001.zip › Figure S2.jpeg]

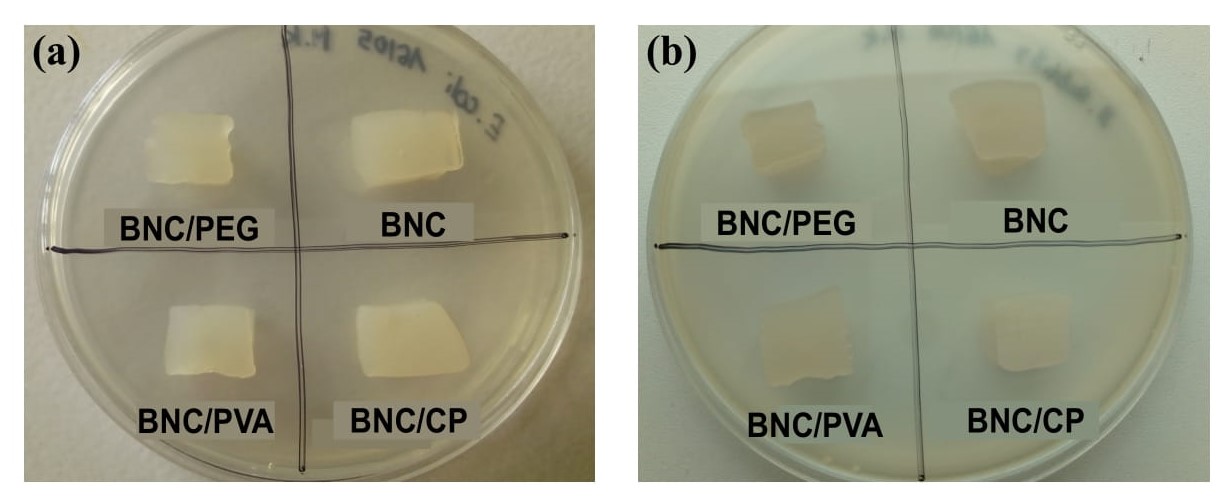

Supplement: Supplementary file 1 [file ijms-23-03391-s001.zip › Figure S3.jpeg]

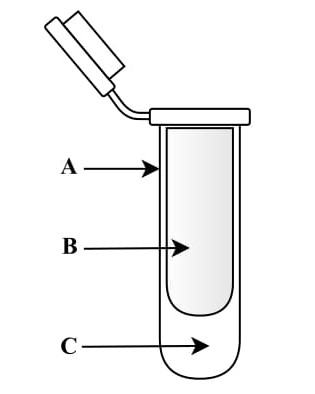

Supplement: Supplementary file 1 [file ijms-23-03391-s001.zip › Figure S4.jpeg]

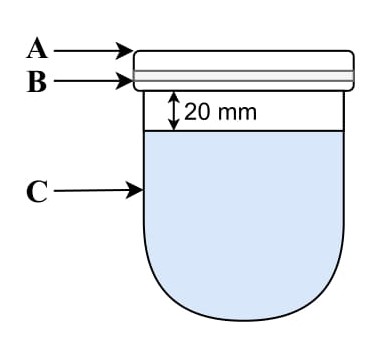

Supplement: Supplementary file 1 [file ijms-23-03391-s001.zip › Figure S5.jpeg]
